# Active thymus in adult with lung cancer: preliminary results from the Adult Thymic Project

**DOI:** 10.1007/s13304-024-01953-w

**Published:** 2024-08-21

**Authors:** Simona Sobrero, Enrico Patrucco, Francesca Napoli, Roberta Ragazzini, Rachele Milazzo, Federico Vaisitti, Chiara Ambrogio, Paola Bonfanti, Ottavio Rena, Enrico Ruffini, Luisella Righi, Francesco Leo

**Affiliations:** 1 Thoracic Surgery Division, Department of Oncology, S. Luigi Gonzaga Hospital, University of Torino, Regione Gonzole 10, Orbassano, 10043 Turin, Italy; 2 Department of Molecular Biotechnology and Health Sciences, Molecular Biotechnology Center, University of Torino, Turin, Italy; 3 Pathology Unit, Department of Oncology, S Luigi Hospital, University of Torino, Turin, Italy; 4 Epithelial Stem Cell Biology and Regenerative Medicine Laboratory, The Francis Crick Institute, London, UK; 5 Thoracic Surgery Division, Ospedale Maggiore della Carità, Novara, Italy; 6 Thoracic Surgery Division, Department of Surgical Science, Azienda Ospedaliera Universitaria Città della Salute e della Scienza di Torino, University of Torino, Turin, Italy; 7 Department of Health Science, Università del Piemonte Orientale, Novara, Italy

**Keywords:** Thymus, Lung cancer, Aging

## Abstract

Thymus is considered a non-functional remnant in adults, but some evidence suggest that it may harbor residual activity. Lung cancer patients represent the ideal model to study thymic residual activity, as their thymus can be easily harvested during surgery. This study was designed to confirm the presence of residual thymic activity both in adult mice (step 1) and in humans (step 2). In step 1, lung cancer was induced by activating k-ras mutation in a cohort of 20 young and adult mice. After killing, thymus and lungs were analyzed. Thymus was considered active when medullary was evident, cortico-medullary ratio was 50:50 or higher and adipose involution was present. In step 2, a cohort of 20 patients, undergoing surgery for lung cancer, had biopsy of pericardial fat pad, site of ectopic thymus. Thymus was considered present if Hassall’s bodies were detected. In mice, active thymus was detected in a high proportion of cases, without significant difference between adult and young (70% vs 44.4% respectively). Two cases without evidence of lung tumor had a fully functional thymus. In humans, ectopic thymus was detected in the pericardial fat pad in 2 cases (10.5%), confirmed by immunohistochemistry. Signs of previous thymic activity were detected in 8 additional patients. Results confirmed thymus activity in animal models and humans with lung cancer, providing the rationale for future systematic mediastinal thymic biopsy. The comprehension of interactions between thymus, lymphocytes and tumor may open a new potentially targetable perspective in lung cancer.

## Introduction

In adults, thymus is considered a non-functional remnant [1]. Nevertheless, several observations stand against this dogma: (1) in ancient autoptic series, Hassall’s bodies were detected in the anterior mediastinum even after the sixth decade, with a preserved cortical- medullary ratio in patients with tumors [2], (2) thymic tumors have a peak of incidence in the 7th decade of life [3] and arise from thymic epithelial cells, confirming that they can persist in adulthood. (3) Even in mice, adult thymus may develop tumors in 10% of cases when k-ras mutation is present [4, 5].

If confirmed, the biological significance of persistent thymic activity remains unclear. Nevertheless, the removal of thymus in adults doubles the risk of developing second primary tumors [6], suggesting that the thymus protects against cancer maintaining an adequate production of CD4 + and CD8 + lymphocytes. Therefore, it can be argued that restoration of thymic function may boost immunity against cancer cells and potentially represents a target of treatment but an effective tool to assess thymic output is lacking.

In fact, measuring thymic activity in adults is difficult [7]. CT and PET scan identify only gross thymic changes when thymic rebound occurs, usually before the age of 40 [8, 9]. As thymic recover is crucial after human hematopoietic stem cell transplantation, in this setting, recent thymic emigrants (RTE) have been proposed as peripheral markers of thymic activity. Unfortunately, these markers do not take into account the issue of peripheral T cell proliferation and consequently thymic output may be misinterpreted [10, 11].

Lung cancer represents the ideal model to study persistency of thymic activity for several reasons. First, it usually occurs over the age of 50. Second, from the anatomical point of view, the thymus is contiguous to the lung and it is technically simple to take thymic biopsy at the time of lung surgery. Lastly, tumor T-cell infiltration is a prognostically relevant process in non-small cell lung cancer [12] and the thymus could play a regulating role in this mechanism.

The Adult Thymic Project (ATP) was designed to confirm the persistence of thymic activity in adults and to investigate its relationship with lung cancer. The aim of ATP step 1 and 2 was to search for adult thymic activity both in animals and humans with lung cancer.

## Methods

The Adult Thymic Project was designed in 2020 in order to investigate the role of the thymus in lung cancer patients. The pilot part of the study was designed to confirm the hypothesis that active thymus is still present in adult individuals affected by lung cancer both in an experimental (step 1) and human (step 2) model.

### Step 1 (Mice model)

As in humans, thymic function decreases with age in mice and sign of thymic involution such as cortical thinning and coalescence of medullary islands are evident in mice aged of 4 weeks or more [13]. The hypothesis of our experiment was to test whether difference in thymic function between young and adult mice still persists after inducing lung cancer. The cohort was composed of 20 Kras + /LSG12Vgeo strain mice carrying a conditional Kras G12V mutation [14]. Tumors were induced by a single intratracheal instillation of Ad-Cre, a viral vector derived from adenovirus carrying Cre recombinase gene which is capable of inducing genetic recombination in lung alveolar cells. The cohort was divided into two groups according to their age at the time of tumor induction, adult mice (aged 8 weeks or more, group A, *n* = 10) and young mice (ages less than 8 weeks, group B, *n* = 10). At the time of killing (8–16 months after tumor induction), thymic and pulmonary samples were fixed in 10% formalin and trimmed along the length of both lobes in order to provide standardized longitudinal sections.

Thymus was defined as active when all these three parameters were present: (a) evidence of medullary portion, (b) cortico-medullary ratio of 50:50 or higher, (c) absence of adipose involution [15]. Lung tumor was classed as single, multiple (< 5 lesions) or diffuse (5 or more lesions). Mice were kept, managed, and killed at the Molecular Biotechnology Center (MBC) Animal Facility of the University of Torino, according to current European (2007/526/CE) legislation in accordance with the guideline for Ethical Conduct in the Care and Use of Animals as stated in The International Guiding Principles for Biomedical Research Involving Animal. The mice were housed in cages with adequate space, bedding material for comfort and maintained under specific pathogen-free conditions, maintaining 12-h dark/light cycle. All experiments were approved by the Italian Health Minister (authorization *n*° 1227/2020-PR).

### Step 2 (Pericardial fat pad biopsy in lung *cancer* patients)

In humans, ectopic thymic tissue may be found at the level of pericardial fat pad in 5–13% of cases [16–18]. When a lung cancer is resected, this adipose tissue is often used for bronchial stump protection, and it can be biopsied during the procedure without any additional harm for the patient. ATP Step 2 was designed to search for residual ectopic thymic activity in pericardial adipose tissue in a series of 20 consecutive patients undergoing lobectomy for lung cancer after obtaining their informed consent. Biopsy was obtained during tissue harvesting before fixation to the bronchial stump with the aim to obtain a sample volume of at least 1 cm^3^. After hematoxylin/eosin (H&E) stain, pathologist reported the presence of thymus in pericardial adipose tissue when epithelial and lymphocytic nests harboring Hassall bodies were identified [19]. Cells were further investigated by immunohistochemistry in order to identify epithelial cells (PanK), thymocytes (TdT), T cells (CD5), B cells (CD20), histiocytes and macrophages (CD 68). The study was approved by the Ethical Committee (n 39/2022 Joint EC S Luigi Hospital, ASLTO3, TO4 and TO5) and conducted at the Thoracic Surgery Division of S Luigi Gonzaga Hospital, University of Turin, Italy, in cooperation with the Pathology Division of the same Institution and the Epithelial Stem Cell Biology and Regenerative Medicine Laboratory of the Crick Institute, London, UK.

## Results

### Step 1 (Mice model)

**Fig. 1:**
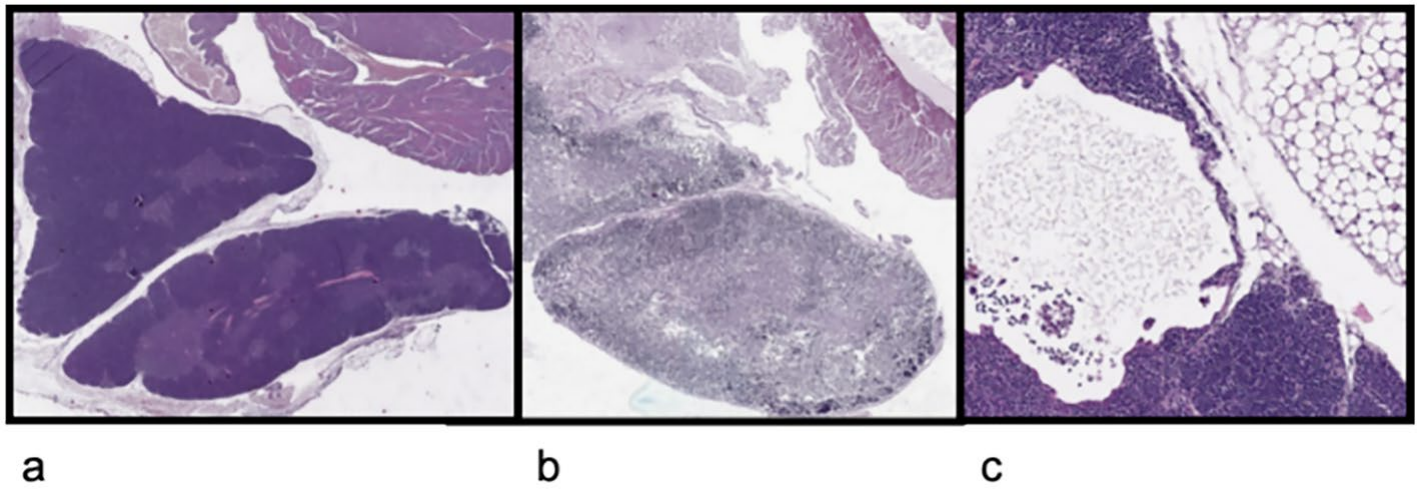
Histological findings of thymus in mice. **a** Mouse 17KY7bis. The presence of active thymus in the oldest mouse; **b** mouse 11KYP12. Atrophic thymus; c. mouse 5KY24bis. Cystic lesion in thymus

In one mouse, the specimen was inadequate. The presence of thymus was detected in 7 adult (70%) and in 4 young mice (44.4%, Table 1, Fig. 1). In the 3 oldest mice (age at inoculation 4.8 months) and killed late in the experiment (16 months after tumor induction), thymus was identified in only one case (33%). The most common event in non-active thymus was the inversion of the cortico-medullary ratio (in 30% of adult mice and 50% of young mice, respectively), followed by adipose involution (20% and 30%, respectively) and absence of medullary (20% in both groups). In adults’ group, induced tumor was classified as single in 3 cases, multiple in 2 cases, diffuse in 5 cases; in young group, 4 cases had multiple tumors and 4 diffuse diseases; no evidence of tumor was detected in 2 cases, both with concomitant normal thymus.

**Table 1:** Thymic findings in mice according to their age of kRAS mutation inoculation

Mice	Sex	Age at inoculation (months)	Medullary portion ±	C:M ratio*	Adipose involution
16KYP12	Male	1.2	+	80:20	No
28KYP13	Female	1.2	+	60:40	No
11KYP12	Male	1.2	+	Atrophic	Yes
7KY23	Male	1.5	+	60:40	No
2KY23 bis	Female	1.5	–	ND	Yes (cystic lesion)
4KY23 bis	Female	1.5	+	50:50	No
5KY24 bis	Male	1.5	+	30:70	No
7KY7 bis	Female	1.7	+	50:50	Yes
6KY43°	Female	2	+	30:70	No
1KY19	Male	2.2	+	90:10	No
4KY20	Female	2.2	+	40:60	No
6KY19	Male	2.2	+	80:20	No
5KY20	Female	2.2	+	60:40	No
3KY42	Male	2.6	+	60:40	no
4KY42	Male	2.6	+	60:40	No
2KY42	Male	2.6	+	60:40	No
17 KY7 bis	Female	4.8	+	80:20	No
3KY7bis	Female	4.8	–	ND	Yes
25KY7 bis	Female	4.8	–	ND	Yes

°control

^*^cortical: medullary ratio (positive for the thymus presence when equal or greater than 50:50

### Step 2 (Pericardial fat pad biopsy in lung *cancer* patients)

**Fig. 2:**
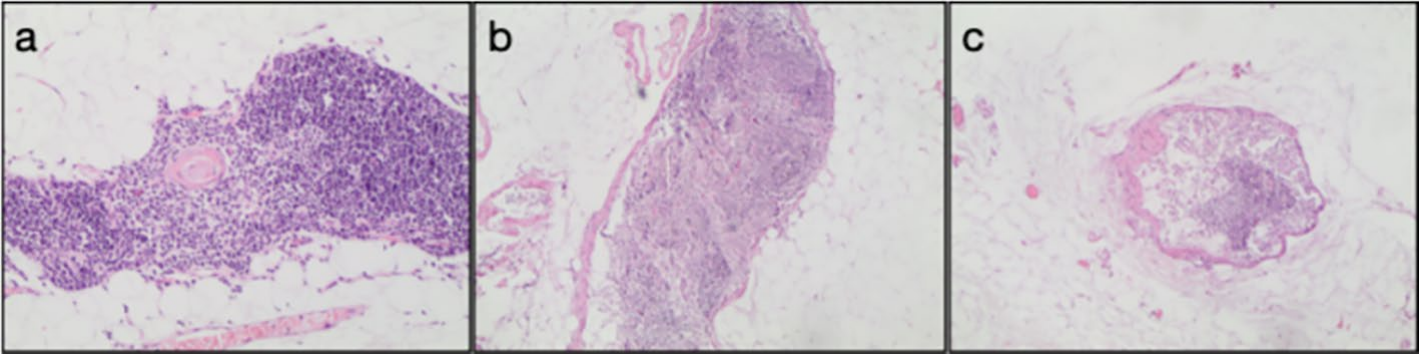
histological findings in pericardial fat tissue in adult lung cancer patients. **a** Hassall’s bodies in pericardial fat tissue; **b** lymphatic nets; **c** cystic lesion

The cohort was composed of 20 patients who underwent resection for lung cancer between April and May 2022. Median age was 67 years and 2 cases had received neoadjuvant chemotherapy before surgery. All patients underwent lobectomy and systematic mediastinal dissection, by open approach in 11 cases and by mini-invasive approach in the remaining cases. Pathological analysis was conducted on 19 cases as one specimen was inadequate (Table 2). At H&E analysis, the presence of thymus (defined by the presence of Hassall’s bodies + lymphatic aggregates) was detected in the pericardial fat pad in 2 cases (10.5%), in which immunohistochemistry confirmed the presence of thymocytes (TdT +). Additional structures potentially related to previous thymic activity were identified in 8 more patients (cystic lesions in 4 patients and isolated lymphatic nests in 4 cases, Fig. 2), with a pattern of chronic inflammation (CD5 + and CD68 +) in 3 cases and a cystic lesion with epithelial lining (panK +) harboring a nest of B cells (CD20 +) in one case. Patients with signs of present or previous thymic activity in the pericardial fat pad were more likely to have a more advanced (≥ IIA) cancer as compared to patients without those signs (50% and 11.1%, respectively, *p* 0.003).

**Table 2:** Description of patients with lung cancer who underwent pericardial fat tissue biopsy according with thymic status

Patient	Sex	Stage°	Hassall’s bodies ±	Cystic lesion	Nests lymphocyte	TDT ±
1	Female	IIIA	–	+	–	–
2	Male	IB	–	–	–	–
3	Male	y(IB)	–	–	+	–
4	Female	y(IIIA)	–	–	+	–
5	Male	IIIA	–	–	–	–
6	Male	IA2	–	–	–	–
7	Female	IA3	–	–	–	–
8	Male	IIB	+	–	–	+
9	Female	IA2	–	–	–	–
10	Female	IIB	–	+	–	–
11	Female	IA3	–	+	–	–
12	Female	IA2	–	–	–	–
13	Male	IIIA	–	+		–
14	Female	IIB	–	–	+	–
15	Male	IA3	–	–	–	–
16	Female	IIIA	–	–	+	–
17	Male	IA2	–	–	–	–
18	Female	IB	–	–	–	–
19	Male	IA1	+	–	–	+
20	Male	IIA	NA	NA	NA	NA

°(*p*) stage: according to the TNM 8° ed

## Discussion

Results from ATP Steps 1 and 2 study proved that the thymus may be functional in adults with lung cancer. In the animal model, like in humans, thymic function reduces over time [13]. Therefore, the finding of our study that adult mice had an active thymus more frequently than young mice was unexpected. If confirmed, this observation suggests that thymic activity depends on the age at the time of tumor induction, being the mature immune system of adults more prone to react. As a corollary, the presence of thymus would represent an activation due to the tumor more than a persistent activity. In addition, this activity does not seem limitless, as in older mice thymic activity was more than halved. Some other observations are speculative but of some interest. For example, having an active thymus seemed associated in this series with a more favorable outcome, as in the two mice who did not develop lung tumor or in the 3 cases who developed a single lesion.

In humans, potential relationship between thymus and lung cancer is not an active research line, despite the increasing importance of immunotherapy in this domain. As a consequence, whether the thymus is active or inactive in lung cancer patients was an unanswered question. The only tool for a definitive answer was a histological proof by thymic biopsy but it was felt unethical due to the total lack of preexisting data. In ATP step 2, the problem was by-passed by harvesting pericardial fat pad, which is a usual location of ectopic thymic tissue and was, therefore, considered as an underestimating proxy. This strategy proved the principle that, in adults with lung cancer, the thymus could be functional in some cases. By serendipity, it was also found that signs of present or past thymic activity were more frequent in advanced stage, suggesting that the cardiophrenic angle area may act as an interface between the lung and the anterior mediastinum and that the tumor may overcome immune response when thymic activity is exhausted. This hypothesis, if confirmed, insert the thymus in the list of potential targets in lung cancer treatment.

This study presents several limits.

In Step 1, two aspects deserve consideration: the limited dimension of the cohort and the choice of controls. The decision to analyze 20 cases was due to the need of background data for further investigations, in which the choice of controls should be probably refined. In fact, comparing thymus in induced and non-induced adult mice is potentially more informative than comparing adults with young induced mice, in which thymus is not mature. This limit is present also in Step 2, as in that part controls are lacking, making difficult to establish whether detected thymus represents persistency or is a response to the presence of the tumor. The other major limit of Step 2 is choosing pericardial fat pad as a site of ectopic thymus, even if its relationship with the thymus in the anterior mediastinum is still unclear. In order to overcome these limits, Step 1 and 2 constitute the rationale for ATP step 3, in which thymic and pericardial biopsy will be routinely performed in lung cancer patients as in controls aged 40 or more undergoing thoracic surgery for non-oncological reasons.

## Conclusions

In conclusion, the thymus is active in adults with lung cancer, potentially opening a new targetable strategy in the management of these patients, as anterior mediastinum, pericardial fat and the lung may represent an interconnected system directly affecting tumor microenvironment.

## Data Availability

Authors agree to make data and materials supporting the results or analyses presented in their paper available upon reasonable request after Ethical Committee approval.
